# Probing the Functionality of Physically Modified Corn Flour as Clean Label Thickening Agent with a Multiscale Characterization

**DOI:** 10.3390/foods9081105

**Published:** 2020-08-12

**Authors:** Alessandro Carcelli, Erica Masuelli, Agoura Diantom, Elena Vittadini, Eleonora Carini

**Affiliations:** 1 Department of Food and Drug, University of Parma, Parco Area delle Scienze 47/a, 43124 Parma, Italy; alessandro.carcelli1@studenti.unipr.it (A.C.); erica.masuelli@studenti.unipr.it (E.M.); 2 Ecole Supérieure des Techniques Biologiques et Alimentaires, University of Lome, BP 1515 Lome, Togo; josephagouradiantom@gmail.com; 3 School of Biosciences and Veterinary Medicine, University of Camerino, via Gentile III da Varano, 62032 Camerino, Italy; elena.vittadini@unicam.it

**Keywords:** corn flour, clean label, physical modification, multilevel method, molecular mobilities

## Abstract

A multilevel and multianalytical approach, combining both traditional and unconventional analytical tools, was used to characterize two physically modified (heated and heated-extruded) corn flours to be used as a “clean label” food ingredient. Physical treatments decreased the resistant starch content and increased the water holding capacity and water binding capacity, more extensively in the product subjected to heating-extrusion, as compared to an untreated control. Heated-extruded flour had the highest ability to form homogeneous systems in cold water while all modified flours produced homogeneous systems when mixed with hot water. Systems made with heated-extruded flour were “more rigid” than other samples at all levels of investigation as they were harder (macroscopic) and had higher storage modulus (mesoscopic), as well as lower proton ^1^H mobility (molecular). Overall, the results highlighted the ability of the multiscale method to give a thorough overview of the flour–water interactions and showed highest water affinity of heated-extruded flour. Heated-extruded flour was then tested in three real-food industrial applications (carrot soup, tomato sauce and a meat patty), where it was successfully implemented as a clean label thickening agent.

## 1. Introduction

Currently, consumers are very interested in the quality of food they eat and are turning in favor of products considered more “natural”, “healthy” and “familiar”. These choices are leading the food industry to develop “clean label” food products with a limited number of ingredients, free from E-numbers additives and/or from substances consumers are not acquainted with [1]. The food industry is therefore pushed to search for new natural ingredients with comparable technological functionality to E-number additives. Starches and flours are commonly used by the food industry as thickening and gelling agents. These ingredients show a limited functionality in their native form; for this reason chemical and physical modifications are usually performed to improve and modulate their functionality. Ingredients that have undergone chemical (crosslinkings, hydroxypropylation, phosphorylation, etc.) modifications need to be labeled as E-numbers, on the basis of EU Regulation 1333/2008 on food additives. On the contrary, flours subjected to physical modification must not be labeled with E-numbers and represent a way to fulfill the demand for a clean functionalization of ingredients. Heat treatment and extrusion processes are two types of the physical modifications used by the industry to obtain pregelatinized starch and flours. Starch gelatinization occurs during heat treatment and extrusion processes that alter the paracrystalline structure of starch granule, increase the exposure of hydroxyl groups of amylose and amylopectin and the portion of starch in the amorphous state, facilitating and ameliorating the interaction with water and, therefore, inducing the starch thickening effect [2,3,4,5]. Anyway, the use of flour should be preferred over the use of starch because of its lower cost of production for the absence of the starch extraction phase. Pregelatinized flour has been tested as clean label thickening agents in bread [6], in reduced-fat mayonnaise [7] and in sauces [8].

A multiscale approach to highlight and an in-depth understanding of the effect of physical modification on functional properties of flours at the macroscopic, mesoscopic and molecular level may be of high interest.

Traditional techniques used for the characterization of flours/starch usually focus on their macroscopic and mesoscopic properties, in particular studying their hydration (e.g., moisture content, water binding capacity and water holding capacity), pasting (viscosity during heating cycle), thermal (differential scanning calorimetry) and viscoelastic (rheometer) properties [9,10,11,12,13,14,15]. Unconventional techniques could be useful to better elucidate, clarify and understand changes occurred at the molecular level determining the macroscopic and mesoscopic functionalities, which are detected by the traditional techniques. The effectiveness of ^1^H low resolution NMR spectroscopy to study the protons mobility and dynamics at the molecular level in food systems has been well reported. In fact, this technique is able to detect different protons populations related to different environments characterized by different molecular mobilities. Polymers in flour interact with water altering its mobility and dynamics and ultimately affecting rheological and physicochemical functionality of products. Different studies have already been performed on starch and flour model systems to assign the different protons domains to different components of flour biopolymers [16,17,18,19,20].

In this perspective, the aim of this study was the evaluation of the technological functionality of physically modified corn flours using a multiscale investigation at the macroscopic, mesoscopic and molecular level, as compared to a native corn flour. Additionally, the thickening properties of the most performing flour were tested as a clean label ingredient in real food applications.

## 2. Materials and Methods

### 2.1. Materials

A native corn flour used as the control sample (CWF14) and two corn flours obtained with two different physical treatments (CWPF14 and HI-MODI FLOUR M) were obtained from local producers (CWF14 and CWPF14 by MartinoRossi (Cremona, Italy) and HI-MODI FLOUR M by HI-FOOD (Parma, Italy)). All flours had the following granulometry (>355 µm 0–2%, >250 µm 0–6%, >150 µm 55–65%, <150 µm 20–30%) and a similar nutritional profile (carbohydrate 80%, proteins 6%, lipids 1% and fiber 1.5%), as reported in their technical data sheets.

All flours were obtained starting from white corn horny grits (*Zea mays* L.). Corn grits were milled to obtain native flour (C) or pretreated, dried and then milled, to obtain physically modified samples, as described in the following. Grits pretreatments consisted of heating or a combination of heating and extrusion processes and were carried out by the MartinoRossi S.p.a. company (Cremona, Italy). The heat treatment was carried out using a conventional steam cooker (CVB, Ocrim, Italy) operating at temperature = 100 °C, pressure = 1.03 bar and time = 120 min on grits at 30% moisture content (g water/100 g of sample); this sample was named M_1_. The combined heating and extrusion treatment was carried out using a twin screw extruder with a length to diameter ratio of 20:1 (HMR, Ocrim, Italy) operating at temperature = 90 °C, pressure = 5 bar and time = 30 min at 30% moisture content of the grits; this sample was named M_2_. M_1_ and M_2_ were subsequently dried to ≈13% moisture content and milled in a roller mill (MPI, Ocrim, Italy) at 25 °C.

### 2.2. Flours Physicochemical Characterization

#### 2.2.1. Amylose Content

The amylose content of the flour samples was determined using the Megazyme Amylose/Amylopectin test kit K-AMYL 12/16 (Megazyme International Ireland Ltd., Co. Wicklow, Ireland) according to the assay procedure, which is based on a modification of the Con A method [21]. Three replicates were performed for each sample.

#### 2.2.2. Resistant Starch Content

Resistant starch (RS) content was measured in accordance to the AOAC method 2002.02 [22] using the Megazyme resistant starch test kit (Megazyme International Ireland Ltd., Co. Wicklow, Ireland) according to the assay procedure. Three replicates were performed for each sample.

#### 2.2.3. Differential Scanning Calorimetry (DSC) Measurement

Flours thermal properties were studied using a differential scanning calorimeter (DSC-Q100 TA instruments Waters, New Castel, DE, USA). The instrument was calibrated with indium (melting temperature: 156.6 °C, melting enthalpy: 28.71 J/g) and mercury (melting temperature: −38.83 °C, melting enthalpy: 11.44 J/g). Briefly, 30 g of corn flour were mixed with 90 mL of distilled water and equilibrate overnight at room temperature. Wet flour samples were then weighted (5–10 mg range) and placed into hermetic stainless-steel pans (PerkinElmer, Waltham, MA, USA) and heated from 10 to 100 °C at a rate of 5 °C/min. Thermal properties To (onset transition temperature), Tp (peak transition temperature), Te (end transition temperature) and ΔH (transition enthalpy, J/g sample) were obtained from the thermograms using the Universal Analysis Program (Version 1.9 D; TA Instruments, New Castle, DE, USA). At least three measurements were performed for each sample.

#### 2.2.4. Flour Hydration Properties

Water holding capacity (WHC, defined as the amount of water retained by the sample without being subjected to any stress) and the water binding capacity (WBC, defined as the amount of water retained by the sample under low-speed centrifugation) of corn flours were measured using the method described by Sarangapani and coworkers with slight modifications [14]. To measure WHC, corn flour samples (2.00 ± 0.05 g) were mixed with distilled water (20 mL) and kept at room temperature for 24 h in vials covered with Parafilm^®^ to avoid water evaporation. The supernatant was decanted with vacuum pump and filter paper to assure that all water was collected. WHC was expressed as grams of water retained per gram of solids. Measurements were taken in triplicates. To measure WBC, corn flour samples (2.00 ± 0.05 g) were mixed with distilled water (20 mL) and centrifuged at 2000× *g* for 10 min with centrifuge ALC PK121R (A.L.C. International S.r.l., Milano, Italy. WBC was expressed as grams of water retained per gram of solids. Measurements were obtained as the average of three replicates.

### 2.3. Multiscale Characterization of Flour–Water Systems

#### 2.3.1. Flour–Water Systems Preparation

Flour–water systems (C, M_1_ and M_2_) were prepared at different flour:water ratios (1:3, 1:4, 1:5, 1:6, 1:7, 1:8 and 1:9) in cold (CC, water temperature 25 ± 2 °C) and in hot conditions (HC, water temperature 100 ± 2 °C) to evaluate the range of flours functionality in a water-based system. Water and flour were placed in a mixer equipped with a whisk (Artisan, Kitchen Aid, Benton Harbor, MI, USA) and mixed at 135 rpm for 2 min. The obtained systems were then placed into aluminum molds and stored overnight at room temperature prior to being analyzed. Two batches of each system were produced in two different days.

#### 2.3.2. Macroscopic Characterization

##### Water Activity and Moisture Content

Water activity (*a_w_*) was measured at 25 °C with an Aqualab 4 TE (Decagon Devices, Pullman, WA, USA). Moisture content (MC, g of water/100 g of sample) was measured by weight loss by drying in a forced-air oven (ISCO NSV 9035, ISCO, Milan, Italy) at 105 °C to constant weight. At least three measurements were taken for each system for a total of six determinations for both parameters.

##### Bostwick Running Distance and Texture Analysis

Bostwick running distance was tested with a Bostwick consistometer (LS100, Laboscientifica, Parma, Italy). The Bostwick consistometer chamber was filled with samples and the distance (cm) travelled after 30 s from the release of the gate of the chamber, was recorded. Three measurements were performed for each system for a total of six determinations.

Hardness (maximum compression height of the peak, N) was performed using a TA.XT2 Texture Analyzer (Stable Micro Systems, Godalming, UK) using a spherical probe P/1SP. Samples were placed in an aluminum mold (85 mm wide and 40 mm height) and analyzed at room temperature after overnight rest. The probe penetrated into the sample to 10% strain at a rate of 2 mm/s. At least five measurements were performed for each sample for a total of at least ten determinations.

#### 2.3.3. Mesoscopic Characterization

##### Rheological Properties

Rheological properties of the different systems were determined using a controlled stress rheometer (Anton Paar MCR 702 twin drive) at 25 °C with a 50 mm diameter plate–plate geometry and a gap of 1 mm. The linear viscosity range (LVR) was determined with a preliminary strain sweep test at 10 Hz. Viscoelastic properties were studied using a frequency sweep test from 0.1 to 10 Hz at 25 °C applying a constant strain of 0.2%. Once the gap was taken to test the gap, the sample was trimmed and Vaseline oil was applied to the edges of the samples, which are not in contact with the plate surfaces. After sample loading, sample went through a resting time until axial force reached about 0 N prior to the start of the experiment to allow for temperature equilibration and dough relaxation. The storage modulus (G’), loss modulus (G”) and tan δ (δ = G”/G’) were recorded. Three measurements were performed for each sample for a total of six determinations.

#### 2.3.4. Molecular Characterization

##### ^1^H Molecular Mobility NMR

^1^H molecular mobility was studied with a Low Resolution Nuclear Magnetic Resonance (NMR) spectrometer (20 MHz, the MiniSpec, Bruker Biospin, Milano, Italy) working at 25.0 ± 0.1 °C. Approximately 4 g of sample were placed into an NMR tube (10 mm diameter) and sealed with Parafilm^®^ to avoid water loss during the test. ^1^H free induction decay (^1^H FID) and proton transverse relaxation time (^1^H T_2_) experiments were performed to investigate the mobility of the more and the less rigid protons, respectively. ^1^H FIDs were acquired using a single 90° pulse, followed by a dwell time of 7 μs, a recycle delay from 2 to 7 s, depending on sample relaxation time, a 0.5 ms acquisition window and 32 scans. ^1^H FID relaxation curves were fitted with a two-component model (exponential and Gaussian; [23]) to obtain quantitative information about the relaxation time and percentage of protons belonging to the more rigid and more mobile proton populations detectable within the FID experimental time frame (ranging from 7 to 500 μs). The fitting was performed with the SigmaPlot v.6 software (Systat Software Inc., San Jose, CA, USA) according to the following equation:

$$f=y_{0}+A*e(-\frac{t}{TA})+B*e{\left(-\frac{t}{TB}\right)}^{2}$$

where *y*_0_ is the FID decay offset, *A* and *B* are the intensities of each relaxation component, *TA* and *TB* are the apparent relaxation times.

^1^H T_2_ relaxation time was measured with a Carr–Purcel–Meiboom–Gill (CPMG) pulse sequence with a recycle delay of 5 s, interpulse spacing of 0.04 ms, 32 scans and 15.000 data points. ^1^H T_2_ curves were analyzed as quasi-continuous distributions of relaxation times with the UPENwin software (Alma Mater Studiorum, Bologna, Italy). Default values for all software UPEN parameters were used with the exception of one parameter (LoXtrap) that was set to 1 to avoid extrapolation of relaxation times shorter than the first experimental point. Experimental curves were also fitted with a discrete multiexponential model (Sigmaplot, v.6, Systat Software Inc., San Jose, CA, USA).

### 2.4. Industrial Application

Based on the results of flours and water–flour systems characterization, the most performing flour (M_2_) was selected, and the effectiveness of its technological functionality was assessed in real food. For this purpose, three food products were selected: a carrot soup, a tomato sauce and a meat patty.

#### 2.4.1. Industrial Food Recipes Preparation

(i) Carrot soup: a commercial fresh carrot soup (Zerbinati, Alessandria, Italy) based on water, carrots, potatoes, celery, EVO and salt was used as the standard (STD). Increasing the level of M_2_ flour was added to the standard (1–3%, g of flour/100 g sample; S1–S3 samples) and mixed and heated up to 70 °C for 4 min in a cooking mixer (Thermomix^®^, Vorwerk, Germany). All samples were prepared in duplicate.

(ii) Tomato sauce: an industrial recipe of tomato sauce was used as the standard (STD). Mix tomato puree (Metro Chef, Milano, Italy; 56.1 wt %), water (30.6 wt %), EVO oil (Farchioni, Perugia, Italy; 6.1 wt %), mirepoix (5.1 wt %), basil (1.2 wt %) and salt (0.8 wt %; ESCO, Hannover, Germany) were mixed at 300 rpm and heated up to 90 °C, cooked under vacuum for 15 min at 115 °C in a bowl chopper (Polyfunctional Qbo 8-3, Roboqbo, Italy). On the basis of the standard recipe, three samples with increasing level of M_2_ (1–3%, g of flour/100 g sample; S1–S3 samples) were also prepared in duplicate.

(iii) Meat patty: an industrial recipe of meat patty was used as the standard (STD). Beef minced meat (93.7 wt %), water (5 wt %), salt (1.2 wt %; ESCO, Hannover, Germany) and ascorbic acid (0.1 wt %; Faravelli, Milano, Italy) were mixed in a kitchen machine equipped with a whisk at 120 rpm for 5 min (Major KMM77XX, Kenwood, Treviso, Italy) and subsequently 70 g formed in a burger patty molder. On the basis of the standard recipe, three samples with an increasing level of M_2_ flour (1–3%, g of flour/100 g sample; S1–S3 samples) were also prepared in duplicate.

#### 2.4.2. Food Characterization

Rheological properties of carrot soups and tomato sauces were tested with a Bostwick consistometer (LS100, Laboscientifica, Parma, Italy). The Bostwick consistometer chamber was filled with sample and the distance (cm) travelled by the sample after 30 s from the release of the gate of the chamber, was recorded. Three measurements were performed for each sample.

Cooking yield (CY) of the meat patties was measured on the basis of the method of Madane and colleague [24] by weighting the sample before and after cooking on a pan. CY was obtained in a percentage on the basis of the following equation:

$$Cooking\;yield=\frac{weight\;of\;cooked\;meat\;patty}{weight\;of\;raw\;meat\;patty}\times 100$$

### 2.5. Statistical Analysis

Significant differences (*p* ≤ 0.05) among different samples were assessed by a one-way-analysis of variance (ANOVA) with a Duncan post-hoc test and a Student’s *t*-test using IBM SPSS statistical software (Version 24.0, SPSS Inc., Armonk, NY, USA).

## 3. Results and Discussions

### 3.1. Flours’ Physicochemical Characterization

Flours were analyzed for their amylose and resistance starch (RS) content and relative results are reported in Table 1.

Amylose content of C, M_1_ and M_2_ was found to be respectively ≈27.4%, ≈25.2% and ≈28.3%. These values were in line with common corn starch, which amylose content was reported in the range 25–27% [10,25]. Physical treatments did not affect the amylose content of the three flours, as previously reported in the literature [10,26]. An amylose increase would have been possible only under severe shear degradation processes during extrusion, which would lead to amylopectin fragmentation [27]. A significant effect of the physical treatment was instead found on RS that decreased in M_1_ (≈1.7%) and M_2_ (≈1.9%) if compared with C (≈5.5%). Pre-gelatinization on M_1_ and M_2_ due to the effect of temperature and pressure during physical treatment favor the loss of starch granule integrity and an opening of their crystalline structures making easier the access of hydrolytic enzymes [5,11,26,27,28].

**Figure 1:**
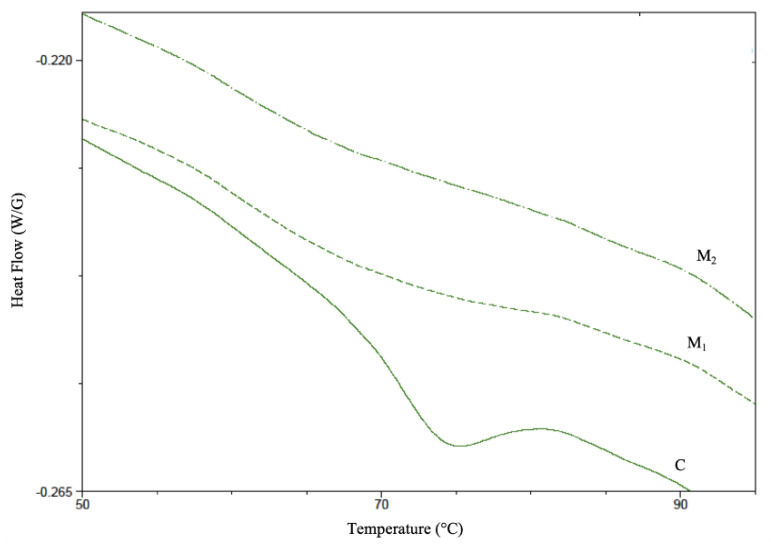
Differential Scanning Calorimetry (DSC) representative thermograms in the 50–95 °C temperature range of the three different corn flours C, M_1_ and M_2_.

Gelatinization process occurred on the grits during the physical treatments were highlighted studying the thermal properties of the different flours by DSC (representative thermograms are shown in Figure 1, while To, Tc, Tp and ΔH are reported in Table 1). One endothermic peak was identified in all samples in the range 60–85 °C and it was attributed to starch gelatinization.

To and Tp of M_1_ and M_2_ samples shifted to significantly lower values if compared to C indicating a structure alteration of native starch that favored the occurrence of gelatinization at lower temperature. ΔH of the C flour was ≈2.3 J/g, and it was found significantly decreased in M_1_ (≈0.8 J/g) and M_2_ (≈0.4 J/g), with significative differences due to the physical treatment applied on grits. The lower gelatinization enthalpy, To and Tp in modified flours as compared to native ones is associated to the physical treatments these samples were subjected to that induced partial starch gelatinization during processing. The combined heating and extrusion physical treatment was found the most effective on the starch gelatinization.

Physical treatments and the consequently pregelatinization enhanced the hydration properties (WHC and WBC) of the flours with M_2_ the flour with the highest water holding and binding capacity. The WHC and WBC increase is associated to the breakage of intra and inter hydrogen bonding, the presence of a less ordered molecular structure and consequently an increase of hydroxyl groups able to bind more water molecules [5,10,12].

### 3.2. Flour–Water Systems

To study the functionality of physically modified flours, flour–water systems were produced considering a wide range of flour:water ratios (1:3, 1:4, 1:5, 1:6, 1:7, 1:8 and 1:9) in the cold condition (CC) and hot condition (HC; Table 2).

An empirical evaluation on systems has been performed to check the absence of water syneresis and the formation of lumps at room temperature for 30 min. Different treatments on flours affected their ability to form homogenous systems avoiding syneresis. In CC, C was not able to form homogeneous systems, as expected. M_1_ was able to avoid water syneresis up to 1:4 while M_2_ up to a 1:5 flour:water ratio. In HC, C was able to avoid water syneresis up to 1:5 while M_1_ and M_2_ up to a 1:9 flour:water ratio. Only the homogenous systems were subsequently analyzed. The higher ability to avoid water syneresis for M_2_ in CC were related to the higher pre-gelatinization level occurred on this sample by physical treatment (as confirmed by thermal properties) and to the consequent higher WHC and WBC. Increasing ability of all flours to interact with water in HC can be attributed to the increased starch gelatinization occurred during samples preparation in the presence of a high amount of water and temperature over ≈60 °C.

### 3.3. Macroscopic Characterization

Moisture contents (MC) and Bostwick running distances are reported in Table 3.

MC of systems increased with the increase of the water level in all flours, as expected, with no significative differences due to the flour treatment applied to grits. For clarity purposes, the subsequent characterization results on systems will be described on the basis of the approximate moisture content of the samples rather than the flour:water ratio. *a_w_* values of systems at all moisture content were higher than 0.99 (data not shown) with no significative differences among systems obtained by different flours, indicating that physical treatment on grits did not affect the water–solids interaction at the macroscopic level.

The Bostwick Running Distance is inversely related to the systems consistency. Bostwick Running Distance increased with increasing moisture content in all samples, due to the plasticizing effect of water that made systems more liquid-like. In both CC and HC, significantly lower Bostwick running distance was observed for M_2_, followed by M_1_ and lastly C. A comparison of systems consistency formed with all flours could be possible only at HC and 83% moisture content; running distance for C, M_1_ and M_2_ was ≈1.6, 0 and 0 cm, respectively.

**Figure 2:**
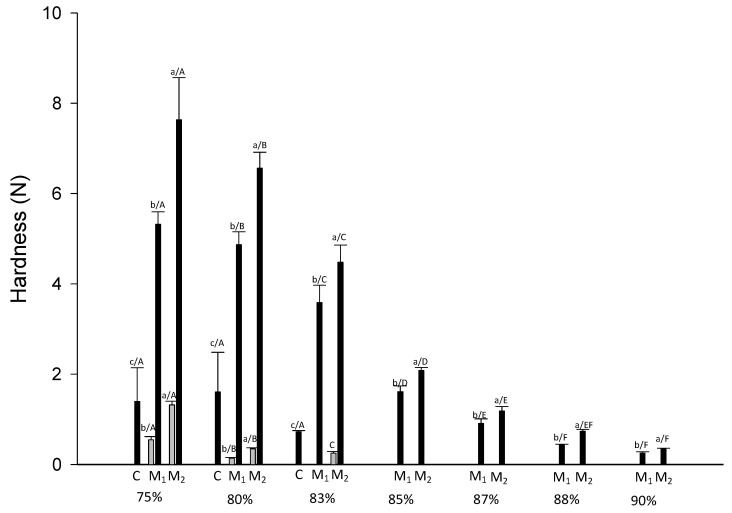
Hardness value of systems formed using three different flours C, M_1_ and M_2_ at different conditions (black HC and grey CC) and different moisture content (75%, 80%, 83%, 85%, 87%, 88% and 90%). Different letters indicate significative differences among samples (*p* ≤ 0.05) where the small letters refer to the differences due to the flours and capital letters refer to the moisture content at the same preparation condition.

Systems textural properties were also analyzed using a texture analyzer by the measure of hardness (Figure 2).

In all studied conditions, hardness decreased with increasing moisture content, as expected, due to the plasticizing effect of water on macroscopic consistency. In the CC and HC systems obtained by M_2_ flour had higher hardness than those obtained by M_1_; where a comparison was possible, systems formed with C had always the lowest hardness value.

In general, flours with higher WBC value led to the formation of systems with lower Bostwick running distance and higher hardness. At HC, the reduced Bostwick running distance and increased hardness can be associated to the increased gelatinization process occurred using hot water and the associated increase of systems viscosity after cooling.

### 3.4. Mesoscopic Characterization

**Figure 3:**
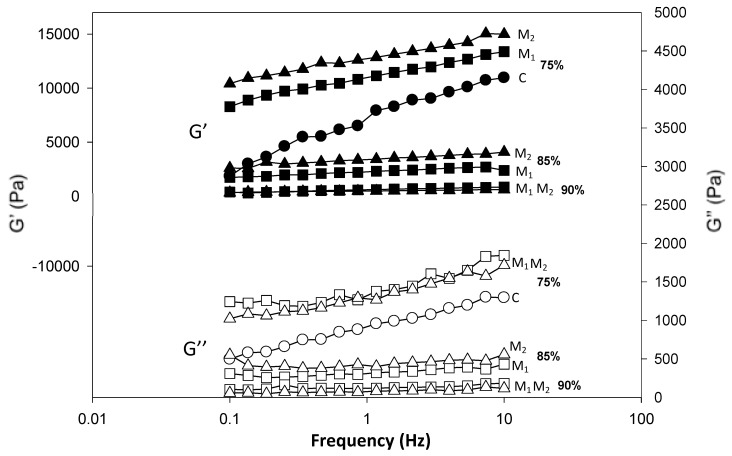
Frequency sweep curves of the systems formed with the three flours (circle: C, square: M_1_ and triangle: M_2_) at three different moisture contents (75%, 85% and 90%) at the hot condition (HC).

**Figure 4:**
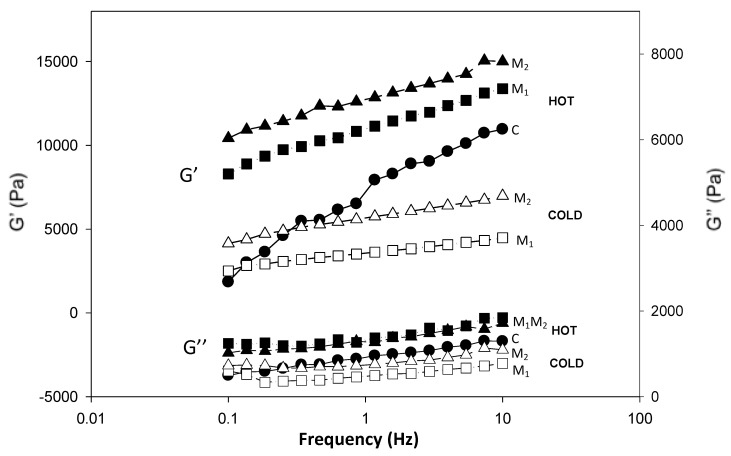
Frequency sweep curves of the systems formed with the three flours (circle: C, square: M_1_ and triangle: M_2_) at the 75% moisture content in the cold condition (white) and hot condition (black).

At a mesoscopic level, homogeneous systems were investigated for their viscoelastic properties. G’ and G” curves versus frequency for samples at selected moisture content (75%, 85% and 90%) at HC are reported in Figure 3, while the effect of water temperature on rheological attributes of the systems is shown in Figure 4 for 75% moisture content samples.

**Figure 5:**
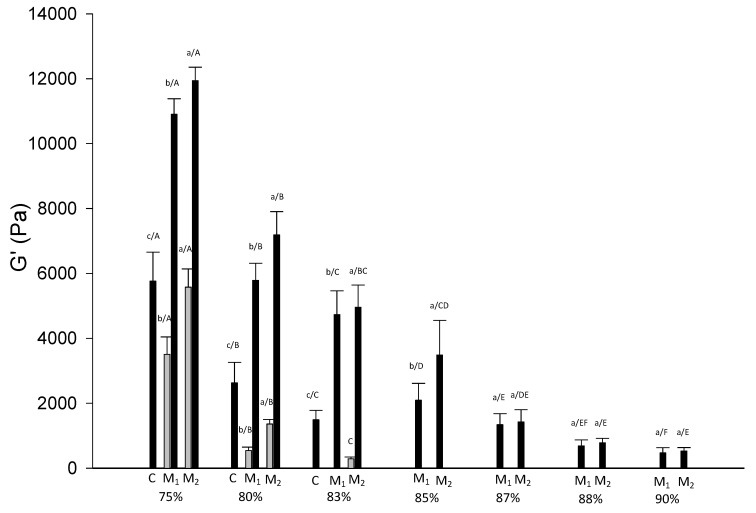
Storage modulus (G’) of systems formed using three different flours, in the CC (grey) and HC (black) and different moisture content (75%, 80%, 83%, 85%, 87%, 88% and 90%). Different letters indicate significative differences among samples (*p* < 0.05) where the small letters refer to the differences due to the flours and capital letters refer to the moisture content at the same preparation condition.

At all moisture contents and temperature conditions the storage modulus (G’) was higher than the loss modulus (G”) in the selected experimental frequency range (0.1–10 Hz) and tan δ < 1 indicating solid-like properties of all systems. G’ and G” were also frequency dependent increasing with the increase of frequency at low moisture contents. With the increase of moisture content, the frequency dependence of G’ and G” was found lower (Figure 3). G’ higher than G” for corn flour systems obtained by heating was also observed by Singh and colleagues [15] and by Rosalina and Bhattacharya, which have analyzed corn starch–water systems [29]. To compare G’ values of different samples, a single frequency 0.858 Hz was selected (Figure 5).

The increase of the moisture content always reduced G’ of the systems formed with the different flours. Furthermore, where a comparison was possible, both CC and HC systems formed with M_2_ had always the highest G’ followed by M_1_ and lastly C. G” followed the same trend of G’ while tan δ had an inverse trend (data not shown). Higher G’ of M_2_, keeping in mind that G’ represents the recoverable energy of systems after deformation, suggested a more rigid structure when the flour used was obtained with the combination of heating and extrusion process. The rheological properties were in agreement with the Bostwick running distances and hardness values obtained at the macroscopic level.

### 3.5. Molecular Characterization

Relaxation times and relative abundances of ^1^H FID populations A (popA) and B (popB) are reported in Table 4.

Pop A relaxed in the range ≈0.017–0.025 ms and represented ≈10–35% of total protons detected in the ^1^H FID frame. Pop A significantly changed as a function of the water content, physical treatment and condition of preparing systems. Pop A was previously attributed to the CH protons of the rigid crystal phase of starch in a corn starch–water system [16]. In this study, the relaxation time and relative abundance of this less mobile Pop A increased and decreased, respectively, with moisture content increase (in both CC and HC) indicating an increase of mobility of these protons due to the plasticizing effect of water on biopolymers. Similar results were observed by Bosmans and colleagues [17] who have observed a decrease of the area of Pop A with the increase of water concentration in samples prepared with potato and rice starch. Comparing samples obtained by different physical treatments at the same moisture content, it could be observed a different trend based on systems prepared in CC or in HC. In fact, Pop A abundance decreased with the increase of strength of physical treatment (M_1_ > M_2_) when systems were prepared in CC, or increased (C < M_1_ < M_2_) when systems were prepared in HC. In CC, the higher mobility of the system (lower abundance of Pop A) in M_2_ than M_1_ was related to the decrease of rigidity of the starch granule due to a higher gelatinization level reached because of stronger physical treatment (as also confirmed by thermal analysis) used on flour. When HC was applied in preparing systems, an additional effect of temperature could have further altered the starch granules integrity. In fact, it can be noticed a higher Pop A% in samples formed in CC than those formed at HC. Same results were noticed by Bosmans and coworkers [16] studying a corn starch–water model system after the heating process at 110 °C for 10 min. If the starch granules integrity was further altered when HC was applied in preparing systems, a higher amount of amylose release from the starch region could be hypothesized. During systems cooling, the released amylose underwent fast recrystallization possibly resulting in a decrease of mobility of these protons (Pop A abundance C < M_1_ < M_2_). Indeed, as also hypothesized by Bosmans and colleagues [16], Pop A% of a starch–water system after the heating process can represent CH protons of amorphous starch immobile after heating and/or newly formed amylose crystals formed during cooling of the sample.

As mentioned before, relaxation time of the more rigid population T_A_ of the systems formed in the CC and HC at all moisture content ranged between ≈0.017 and 0.025 ms with significant but slight differences depending on different samples and therefore no additional discussion was deserved.

Pop B relaxed in the range ≈0.5–0.9 ms increasing its mobility (relaxation time) with moisture content increase while becoming less mobile when flour was subjected to physical treatment (T_B_ significant different as C < M_1_ < M_2_). Pop B % changes were linked to the relative changes of Pop A abundance. Bosmans and colleagues assumed that Pop B represents non-exchanging CH protons in the amorphous part of the granule [16].

Three not well resolved ^1^H populations named population C (Pop C), population D (Pop D) and population E (Pop E) were observed by the ^1^H T_2_ distribution of relaxation times relaxing in the range ≈2–10 ms, ≈33–105 ms and ≈50–290 ms, respectively. ^1^H T_2_ experimental curves were also fitted with a three exponential model and ^1^H relaxation times and relative abundances of the three populations were therefore obtained and are reported in Table 4. Three ^1^H T_2_ populations were also observed in studies conducted by Bosmans and colleagues [16] on the corn starches–water model system relaxing at lower relaxation times than relaxation times we found. This misalignment is probably due to the much higher moisture content of samples studied than those studied by Bosmans and colleagues. Anyway, the assignment of protons to different domains performed by those authors can be used to explain our changes due to experimental variables.

In all samples, Pop E was the predominant population as it encompassed ≈53–86% of the total protons observed in the ^1^H T_2_ experimental window. The mobility and abundance of this population significantly changed as a function of moisture content, preparation condition and physical treatment used on flour. These protons were previously assigned to water protons that exchange with starch hydroxyl protons in the extragranular space in an unheated corn starch–water sample and to hydroxyl protons of starch and water in the gel network containing the granule remnants in a heated corn starch–water sample [16]. Moreover, Pop C was related to the CH protons of amorphous starch in little contact with water and Pop D to contained hydroxyl protons of intragranular water and starch [16].

Relaxation times of the three ^1^H T_2_ populations C, D and E shifted towards higher values with a moisture content increase in all samples, indicating an increase of mobility due to the plasticization effect of water at the molecular level. Considering the effect of the condition used to prepare systems, it could be noticed that samples prepared in CC, had Pop C and D more abundant than systems prepared in HC, that, on the contrary, were characterized by high abundance of Pop E. Taking in mind that protons of Pop E, as reported before, represent the gel network domain, this different behavior could be related to the achievement of a higher gelatinization degree during the preparation of systems in HC, due to the high temperature of water used.

Physical treatment flours significantly affected the ^1^H T_2_ mobility of systems, both in the CC and HC. In particular, major changes can be related to the decrease of T_E_. In fact, the lowest mobility of Pop E was detected in M_2_ samples, followed by M_1_ and lastly by C samples. A decrease of the T_2_ mobility of the Pop E in a wheat flour–water model system after heating has been observed by Luyts and colleagues, which have suggested that the decreased mobility is predominately attributed to the formation of a structured network and consists predominately of mobile exchanging protons of water, starch and gluten [18]. The stronger condition of the physical treatment used to obtain M_2_ flour allowed us to increase starch gelatinization, as also probed by DSC, and explain the overall higher rigidity of these systems. Overall, lower molecular mobility in M_2_ samples was found in agreement to their higher hardness and G’ values.

The molecular results obtained were related with the macroscopic (Bostwick Running Sistance, hardness) and mesoscopic (G’, G”) properties of the systems, confirming the usefulness of ^1^H low resolution NMR spectroscopy as a tool to deeper investigate the physicochemical functionality of ingredients as the physically modified flours analyzed in this study.

### 3.6. Industrial Applications

Physicochemical characterization of flour–water systems showed M_2_ as the flour with the highest thickening ability among samples considered, due to its major ability to interact with water. To prove its feasibility in real complex foods, the flour has been used as a clean label food ingredient in the formulation of (i) carrot soup, (ii) tomato sauce and a (iii) meat patty. These foods were selected as they represent examples of systems to which thickening ingredients are frequently added by the food technologists for different purposes. In fact, in carrot soups no water syneresis and a proper pulpiness is required; the tomato sauce has to not present water–oil syneresis and has to be creamy, while the meat patty has to retain water during cooking showing a proper juiciness. To assess these features industrial familiar analytical tools have been used: a Bostwick consistometer for the carrot soup and tomato sauce systems, and cooking yield for the meat patty. Both analytical tools are used by the food industry for the quick evaluation of food quality parameters [30,31].

**Figure 6:**
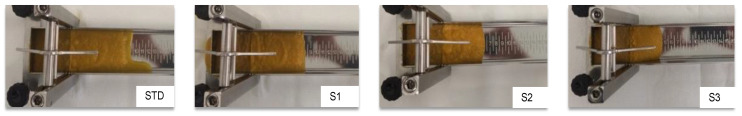
Appearance and Bostwick Running Distances of cooked commercial carrot soup (**STD**) and carrot soup samples at increasing M_2_ level (**S1**–**S3**).

The Bostwick Running Distance (RD) of carrot soup (Table 5) significantly decreased with the increase of M_2_ in the formulation. The overall appearance of the soup also improved with the elimination of the undesired water synesis effect present in the commercial carrot soup, as it can be observed in Figure 6.

**Figure 7:**
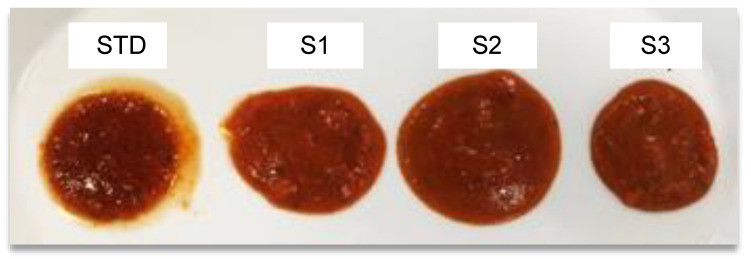
Appearance of tomato sauce (**STD**) and tomato sauce samples at increasing M_2_ level (**S1**–**S3**).

The Bostwick Running Distance of the different cooked tomato sauce samples are reported in Table 5. As reported for the carrot soup the RD significantly decreased with the increase of M_2_. As it can be observed in Figure 7, the overall appearance of the tomato sauce improved as a creamier texture without the presence of undesired water and oil syneresis up to a 2% M_2_ inclusion level. Instead, the S3 sample had a jelly and steady texture, which is undesired in sauces.

For the meat patty, a significant increase of the cooking yield (CY) was observed for S2 and S3 recipe (≈82) compared to STD and S1 (≈78%; Table 5). The CY increase led to an increase of water retention in the patty after cooking and a possible improvement in its juiciness.

Overall, the technological functionality of M_2_ as a thickening agent and water/oil binder was confirmed by the industrial application in real foods. Additional sensorial analysis would be necessary to ascertain the consumer acceptability.

## 4. Conclusions

A multilevel and multianalytical approach was used to study the technological functionality of two physically modified corn flours subjected to different production processes (heating and heating-extrusion, respectively) and a native corn flour. Overall, the results have highlighted the ability of the multiscale method to provide a thorough and global understanding of the occurred flour–water interactions. In particular, differences due to the moisture content, the preparing condition of systems and the physical treatment used on the flour were highlighted at the molecular level and were in agreement with changes showed at the macroscopic (Bostwick running distance, hardness) and mesoscopic (G’ and G”) level. The strengthening of the physical treatment on grits allowed it to further alter the starch native structure towards a more gelatinized structure that was therefore more able to interact with water when flour was used to prepare systems at different moisture content. This modified flour could be therefore used in a wide range of food applications, both in the cold or hot condition. The use in the hot condition allows one to increase the gelatinization degree and therefore to further modulate macroscopic properties, which were driven by molecular changes, as probed before. Particularly important could be the application of this ingredient when a clean label is searched for, as probed by its effectiveness as a clean label thickening agent in real foods.

## Figures and Tables

**Table 1:** Physicochemical properties of three different corn flours (C, M_1_ and M_2_).

	C	M_1_	M_2_
**Amylose (%)**	27.41 ± 4.06 ^a^	25.16 ± 5.27 ^a^	28.32 ± 2.48 ^a^
**Resistant Starch (%)**	5.45 ± 0.01 ^a^	1.74 ± 0.28 ^b^	1.94 ± 0.05 ^b^
**To (°C)**	62.927 ± 2.21 ^a^	57.28 ± 0.42 ^b^	57.55 ± 0.04 ^b^
**Tp (°C)**	4.04 ± 0.57 ^a^	68.49 ± 0.16 ^b^	68.18 ± 0.32 ^b^
**Te (°C)**	84.02 ± 2.37 ^a^	84.54 ± 2.50 ^a^	81.10 ± 0.59 ^a^
**ΔH (J/g)**	2.32 ± 1.09 ^a^	0.84 ± 0.26 ^b^	0.44 ± 0.11 ^c^
**WHC (g/g)**	2.36 ± 0.30 ^c^	4.67 ± 0.20 ^b^	6.44 ± 0.09 ^a^
**WBC (g/g)**	1.65 ± 0.04 ^c^	3.51 ± 0.11 ^b^	4.34 ± 0.13 ^a^

All data are expressed as mean ± standard deviations. Means with different superscript letters for each parameter considered differ significantly (*p* < 0.05).

**Table 2:** Physical appearance of corn flour:water systems subjected to mixing for 2 min at 135 rpm under the cold condition (CC) or hot condition (HC). H means that flour:water systems obtained were homogeneous while S means that flour:water systems showed water syneresis.

Preparation Condition	Flour	Flour:Water Ratio						
		1:3	1:4	1:5	1:6	1:7	1:8	1:9
CC	C	S	S	S	S	S	S	S
	M1	H	H	S	S	S	S	S
	M2	H	H	H	S	S	S	S
HC	C	H	H	H	S	S	S	S
	M1	H	H	H	H	H	H	H
	M2	H	H	H	H	H	H	H

**Table 3:** Moisture content (MC) and Bostwick running distances of corn flour-water systems prepared with three corn flours (C, M_1_ and M_2_) at different flour:water ratio (1:3, 1:4, 1:5, 1:6, 1:7, 1:8 and 1:9) in cold (CC) and hot conditions (HC).

Moisture Content (MC) % (gH_2_O/100 g)			Bostwick Running Distance (cm)
**Cold Condition (CC)**			
M_1_	1:3	75.3 ± 0.6 aB	1.25 ± 0.20 B
	1:4	80.2 ± 0.6 bA	8.42 ± 0.12 aA
M_2_	1:3	75.2 ± 0.1 aC	0
	1:4	80.9 ± 0.5 bB	0.50 ± 0.16 bB
	1:5	83.8 ± 0.2A	4.29 ± 0.29A
**Hot Condition (HC)**			
C	1:3	75.9 ± 0.5 aC	0
	1:4	80.7 ± 0.9 aB	0
	1:5	83.9 ± 0.4 aA	1.58 ± 0.14
M_1_	1:3	74.8 ± 0.5 aF	0
	1:4	80.2 ± 0.2 aE	0
	1:5	83.0 ± 0.6 aD	0
	1:6	85.1 ± 0.8 aC	0
	1:7	87.6 ± 0.3 aB	0.58 ± 0.20 C
	1:8	88.4 ± 0.4 aA	3.54 ± 0.19 aB
	1:9	90.6 ± 0.3 aA	4.21 ± 0.12 aA
M2	1:3	75.2 ± 0.4 aE	0
	1:4	80.6 ± 0.9 aD	0
	1:5	83.1 ± 0.7 aC	0
	1:6	84.9 ± 0.3 aC	0
	1:7	87.2 ± 0.3 aB	0
	1:8	88.4 ± 0.4 aA	0.46 ± 0.19 bB
	1:9	90.3 ± 0.4 aA	2.37 ± 0.14 bA

All the data are expressed as mean ± standard deviations; different letters close to the number indicate significative differences among samples (*p* ≤ 0.05) where the small letters refer to the differences due to the flours and capital letters refer to the water concentration at the same preparation condition.

**Table 4:** Relaxation times (T_A_, T_B,_ T_C_, T_D_ and T_E_) and relative abundances [pop A (%), pop B (%), pop C (%), pop D (%) and pop E (%)] of ^1^H populations (A and B from ^1^H FID, and C, D and E from ^1^H T_2_ experiment) of systems formed with three corn flours (C, M_1_ and M_2_) at different moisture contents (75%, 80%, 83%, 85%, 87%, 88% and 90%) and in the cold condition (CC) and hot condition (HC).

	Moisture Content	Pop A (%)	T_A_ (ms)	Pop B (%)	T_B_ (ms)	Pop C (%)	Tc (ms)	Pop D (%)	T_D_ (ms)	Pop E (%)	T_E_ (ms)
**Cold Condition (CC)**											
**M_1_**	75%	35.12 ± 0.94 aA	0.022 ± 0.000 bA	64.89 ± 0.94 bA	0.49 ± 0.01 aA	7.03 ± 0.54 aB	6.11 ± 0.58 bB	26.86 ± 1.43 bB	33.99 ± 1.38 bB	66.79 ± 0.95 aA	145.04 ± 2.55 aB
	80%	34.49 ± 1.19 aA	0.022 ± 0.000 bA	64.50 ± 1.19 bA	0.51 ± 0.03 aA	10.51 ± 1.75 aA	12.85 ± 1.99 aA	36.76 ± 2.68 aA	76.64 ± 3.64 aA	53.33 ± 0.60 bB	166.71 ± 3.93 aA
**M_2_**	75%	32.10 ± 0.30 bA	0.025 ± 0.001 aA	67.90 ± 0.30 aB	0.46 ± 0.00 bC	6.11 ± 0.30 bB	6.85 ± 0.42 aC	31.81 ± 1.63 aA	38.42 ± 2.20 aC	62.08 ± 1.78 bB	78.49 ± 0.70 bC
	80%	31.42 ± 0.41 bB	0.024 ± 0.001 aB	68.58 ± 0.41 aB	0.49 ± 0.01 aB	6.22 ± 0.78 bB	8.16 ± 1.20 bB	25.94 ± 0.87 bB	43.91 ± 2.78 bB	67.84 ± 1.60 aA	106.06 ± 2.61 bB
	83%	23.28 ± 0.7C	0.024 ± 0.00B	76.72 ± 0.7A	0.56 ± 0.02A	8.09 ± 0.88A	14.05 ± 0.6A	32.80 ± 2.2A	72.32 ± 3.2A	59.35 ± 2.5C	147.14 ± 3.9A
**Hot Condition (HC)**											
**C**	75%	22.81 ± 0.55 cA	0.018 ± 0.000 bA	77.19 ± 0.55 aC	0.69 ± 0.01 aB	4.99 ± 0.38 aA	10.58 ± 0.75 aB	17.25 ± 1.12 b	52.80 ± 2.48 aB	77.76 ± 1.50 aA	122.95 ± 0.87 aB
	80%	20.98 ± 0.59 cB	0.017 ± 0.000 cAB	79.02 ± 0.59 aB	0.70 ± 0.02 aB	4.40 ± 0.86 aA	11.04 ± 0.69 aAB	17.20 ± 0.29 aA	58.50 ± 3.41 aA	78.63 ± 0.93 bA	135.33 ± 3.92 aA
	83%	15.60 ± 1.56 cC	0.017 ± 0.000 bB	84.40 ± 1.56 aA	0.77 ± 0.03 aA	4.08 ± 0.12 aA	12.06 ± 0.46 aA	17.74 ± 0.46 aA	61.17 ± 1.00 aA	78.17 ± 0.42 cA	140.41 ± 3.40 aA
**M_1_**	75%	27.96 ± 1.02 bA	0.019 ± 0.000 bA	72.04 ± 1.02 bE	0.58 ± 0.00 bF	4.50 ± 0.18 aA	5.70 ± 0.34 bF	20.47 ± 1.45 abA	33.18 ± 1.49 bG	75.03 ± 1.62 abE	66.38 ± 0.84 bG
	80%	23.05 ± 0.65 bB	0.018 ± 0.001 bA	77.17 ± 0.79 bD	0.65 ± 0.01 bE	3.62 ± 0.23 abB	7.07 ± 0.98 bE	12.78 ± 1.64 bC	43.35 ± 2.39 bF	83.60 ± 1.87 aC	100.03 ± 2.01 bF
	83%	18.12 ± 1.04 bC	0.019 ± 0.000 aA	81.88 ± 1.04 bC	0.69 ± 0.02 bD	2.85 ± 0.17 bD	8.53 ± 0.19 bD	11.02 ± 0.27 cDE	52.03 ± 1.48 bE	86.13 ± 0.37 aAB	134.41 ± 0.99 bE
	85%	10.38 ± 0.11 bD	0.019 ± 0.000 bA	89.62 ± 0.11 aAB	0.79 ± 0.04 aBC	3.45 ± 0.23 aB	11.85 ± 0.48 aC	15.27 ± 1.81 aB	73.54 ± 1.07 aD	81.28 ± 1.99 bD	150.81 ± 3.08 aD
	87%	10.50 ± 0.20 bD	0.019 ± 0.001 aA	89.49 ± 0.12 aAB	0.78 ± 0.05 aC	3.13 ± 0.08 aC	13.34 ± 0.51 aB	11.96 ± 0.50 aCD	80.15 ± 2.10 aC	84.91 ± 0.51 aBC	197.10 ± 3.69 aC
	88%	10.21 ± 0.70 bD	0.019 ± 0.001 aA	89.79 ± 0.70 aA	0.83 ± 0.03 aAB	2.80 ± 0.10 aD	15.13 ± 0.57 aA	10.17 ± 0.50 bE	86.73 ± 1.49 aB	87.02 ± 0.55 aA	224.75 ± 2.92 aB
	90%	11.54 ± 0.85 bD	0.017 ± 0.002 aB	88.51 ± 0.95 aB	0.88 ± 0.06 aA	2.75 ± 0.05 aD	15.72 ± 0.90 aA	11.54 ± 0.21 bCDE	104.55 ± 1.68 aA	85.71 ± 0.25 aAB	291.32 ± 4.85 aA
**M_2_**	75%	29.07 ± 1.15 aA	0.024 ± 0.001 aB	70.92 ± 1.15 bE	0.48 ± 0.01 cF	3.86 ± 0.28 bA	3.29 ± 0.37 cE	23.89 ± 2.92 aA	24.90 ± 1.87 cG	72.98 ± 2.25 bB	50.18 ± 1.79 cG
	80%	29.24 ± 0.5 aA	0.021 ± 0.001 aC	70.75 ± 0.51 cE	0.50 ± 0.02 cE	2.88 ± 0.31 bBC	3.53 ± 1.29 cE	13.19 ± 0.30 bB	27.79 ± 1.16 cF	83.94 ± 0.17 aA	76.36 ± 1.65 cF
	83%	21.37 ± 1.29 aB	0.025 ± 0.002 aA	78.63 ± 1.29 cD	0.58 ± 0.01 cD	3.07 ± 0.04 bB	7.04 ± 1.44 cD	12.82 ± 1.2 bB	39.23 ± 0.45 cE	84.11 ± 1.16 bA	98.58 ± 3.43 cE
	85%	17.50 ± 0.42 aC	0.023 ± 0.001 aB	82.51 ± 0.42 bC	0.60 ± 0.02 bC	2.85 ± 0.17 bBC	8.53 ± 0.19 bC	11.02 ± 0.27 bB	52.03 ± 1.48 bD	86.13 ± 0.37 aA	134.41 ± 0.9 bD
	87%	14.15 ± 0.96 aD	0.020 ± 0.001 aC	85.85 ± 0.96 bB	0.69 ± 0.02 bB	2.63 ± 0.31 bC	11.75 ± 1.01 bB	14.06 ± 3.99 aB	66.46 ± 1.56 bC	83.97 ± 4.84 aA	149.29 ± 3.15 bC
	88%	11.80 ± 0.22 aE	0.021 ± 0.001 aC	88.20 ± 0.22 bA	0.72 ± 0.01 bA	2.77 ± 0.24 aBC	12.03 ± 0.34 bB	13.01 ± 1.24 aB	72.88 ± 2.44 bB	84.22 ± 1.47 bA	163.93 ± 0.01 bB
	90%	11.73 ± 1.33 aE	0.020 ± 0.001 aC	88.54 ± 0.15 aA	0.74 ± 0.02 bA	2.84 ± 0.13 aBC	14.99 ± 0.35 aA	13.65 ± 1.13 aB	86.33 ± 1.09 bA	83.51 ± 1.26 bA	190.94 ± 5.06 bA

All the data are expressed as mean ± standard deviations; different letters close to the number indicate significative differences among samples (*p* ≤ 0.05) where the small letters refer to the differences due to the flours and capital letters refer to the moisture content at the same preparation condition.

**Table 5:** Bostwick Running Distance of carrot soup and tomato sauce, and cooking yield of a meat patty at increasing M_2_ inclusion level, 1%, 2% and 3%, g of flour/100 g sample.

		M_2_ Inclusion Level			
		STD	S1	S2	S3
**Bostwick Running Distance (cm)**					
	**Carrot Soup**	9.2 ± 0.3 a	7.8 ± 0.3 b	5.8 ± 0.8 c	4.5 ± 0.5 d
	**Tomato Sauce**	15.3 ± 0.6 a	13.2 ± 0.3 b	12.2 ± 0.8 b	8.8 ± 0.3 c
**Cooking Yield (%)**					
	**Meat Patty**	78.6 ± 0.2 b	78.4 ± 0.5 b	82.8 ± 0.4 a	82.7 ± 0.2 a

All the data are expressed as mean ± standard deviations; different letters close to the number indicate a significative difference among the sample (*p* ≤ 0.05) due to the flour inclusion level amount.
